# The role of nasal patency in obstructive sleep apnea: an expert consensus

**DOI:** 10.3389/frsle.2026.1819496

**Published:** 2026-04-28

**Authors:** Andrew M. Vahabzadeh-Hagh, Patrick J. Strollo, Masayoshi Takashima, Kathleen Yaremchuk, Thomas Heineman, Ofer Jacobowitz, Yi Cai, Jolie Chang, Maria Suurna, Carol Yan, Atul Malhotra

**Affiliations:** 1 Department of Otolaryngology Head and Neck Surgery, University of California, San Diego, La Jolla, CA, United States; 2 Division of Pulmonary, Allergy, Critical Care, and Sleep Medicine, Department of Medicine, UPMC Sleep Medicine Center, University of Pittsburgh Medical Center, Pittsburgh, PA, United States; 3 Houston Methodist Hospital/Weill Cornell Medical College, Department of Otolaryngology–Head and Neck Surgery, Houston, TX, United States; 4 Henry Ford Health System, Department of Otolaryngology-Head and Neck Surgery, Division of Sleep Medicine, Detroit, MI, United States; 5 Physicians' Clinic of Iowa, Department of Otolaryngology, Cedar Rapids, IA, United States; 6 New York Eye and Ear Infirmary, Department of Otolaryngology–Head and Neck Surgery, Icahn School of Medicine at Mount Sinai, New York, NY, United States; 7 Columbia University Irving Medical Center, Department of Otolaryngology, New York, NY, United States; 8 Division of Sleep Surgery, Department of Otolaryngology–Head and Neck Surgery, University of California, San Francisco, San Francisco, CA, United States; 9 Department of Otolaryngology–Head and Neck Surgery, University of Miami Miller School of Medicine/UHealth Sleep Center, Miami, FL, United States; 10 Division of Pulmonary, Critical Care, and Sleep Medicine, Department of Medicine, University of California, San Diego, La Jolla, CA, United States

**Keywords:** CPAP adherence, nasal airway obstruction, nasal patency, nasal valve collapse, obstructive sleep apnea, sleep-disordered breathing, temperature-controlled radiofrequency

## Abstract

Obstructive sleep apnea (OSA) is estimated to affect up to 1 billion people worldwide, although the majority of the disease remains undiagnosed and untreated. OSA has major cardiometabolic and neurocognitive sequelae, making effective treatment a public-health priority. Nasal positive airway pressure (PAP) therapy remains the most effective non-surgical therapy, and provides transformative benefits for some patients. However, tolerance and long-term adherence remain problematic for a substantial subset of patients, particularly those with nasal airway obstruction (NAO). Inadequate nasal patency increases resistance and hinders effective PAP use, prompting many patients to struggle with or abandon therapy. This challenge underscores the need to identify strategies that optimize PAP adherence through improved nasal function. A converging body of physiological evidence, large-scale observational data, and controlled clinical trials demonstrates that nasal airway obstruction, particularly at the nasal valve, can be a fundamental limitation to continuous positive airway pressure (CPAP) success. To address this challenge, a multidisciplinary expert panel of clinicians in sleep medicine, otolaryngology, and pulmonology medicine convened to review the physiologic mechanisms linking nasal resistance to upper-airway collapsibility, evaluate outcomes after restoration of nasal patency, and examine the role of minimally invasive therapies such as temperature-controlled radiofrequency (TCRF) remodeling. The group concluded that nasal obstruction management and optimization should be viewed as an integral component of OSA management, with the goal of improving CPAP adherence and patient outcomes.

## 1 Introduction

### 1.1 Nasal airway obstruction (NAO) and obstructive sleep apnea (OSA)

Obstructive sleep apnea (OSA) is estimated to affect up to 1 billion people worldwide (Benjafield et al., 2019), although the majority of cases remain undiagnosed and untreated. OSA carries major cardiometabolic and neurocognitive sequelae, making effective therapy a public-health priority (Punjabi et al., 2009; Redline et al., 2010). Nasal positive airway pressure (PAP) therapy is first-line treatment and provides transformative benefit for many patients (Jenkinson et al., 1999; Sullivan et al., 1981); however, tolerance and long-term adherence remain problematic in a substantial subset.

Nasal airway obstruction (NAO) is a common and under-recognized contributor to PAP intolerance (Chang et al., 2023; Stewart et al., 2004). Compromised nasal patency increases airway resistance and can make nasal PAP therapy difficult to tolerate, leading patients either to switch to oronasal masks that may be less comfortable, require higher pressures, or to abandon therapy altogether. Recognizing this clinical challenge, a multidisciplinary expert panel of otolaryngologists, sleep specialists, and pulmonologists convened to evaluate the current body of evidence, review the physiological basis linking nasal obstruction to sleep disordered breathing, and synthesize consensus guidance on optimizing nasal function to improve PAP adherence and patient outcomes.

The panel discussion focused on synthesizing current evidence regarding nasal airway obstruction (NAO), its physiologic relationship to obstructive sleep apnea (OSA), the influence of nasal resistance on treatment efficacy and adherence, and the evolving role of minimally invasive nasal interventions in clinical management. The panel reviewed published data (presented by AV and OJ), identified existing evidence gaps, and discussed where targeted research could refine patient selection and confirm long-term benefits. Emphasis was placed on consolidating existing evidence rather than proposing new hypotheses, underscoring nasal optimization as a practical and achievable enhancement to standard OSA care. To our knowledge, this work represents the first multidisciplinary expert consensus synthesizing clinical and physiologic evidence on nasal patency and OSA, providing practical guidance to improve CPAP adherence and patient outcomes.

## 2 Methods/panel process

The expert panel was convened by invitation based on clinical and research experience and domain knowledge in sleep medicine, otolaryngology, and pulmonology. All participants reviewed pre-circulated evidence summaries and participated in structured virtual discussions moderated by an academic facilitator. Consensus statements were refined iteratively and approved unanimously by all co-authors.

## 3 Physiologic basis of nasal resistance and sleep disruption

### 3.1 The nasal valve and airflow dynamics

The internal nasal valve, formed by the junction of the upper lateral cartilage, septum, and inferior turbinate, accounts for approximately 50%−60% of total upper airway resistance (Lam et al., 2006; Rhee et al., 2010). According to Poiseuille's law (R ∞ 1/r4), resistance is inversely proportional to the fourth power of radius (Courtiss and Goldwyn, 1983). Induced nasal obstruction converts laminar inspiratory flow into turbulent jets, propagating negative pressure effects through Bernoulli mechanisms and increasing the critical closing pressure (Pcrit). Even a minor 15%−20% decrease in nasal valve radius can markedly increase airflow resistance. Elevated nasal resistance increases negative inspiratory pressures downstream, predisposing to pharyngeal collapse and airway obstruction. Imaging and computational flow studies have demonstrated that nasal obstruction amplifies pharyngeal collapsibility during sleep (Brimioulle and Chaidas, 2022; Suzuki and Tanuma, 2020; Wakayama et al., 2016). This highlights that nasal obstruction is not isolated but functionally coupled to downstream airway behavior, influencing OSA pathophysiology.

### 3.2 Reflex pathways and neuromechanical coupling

Nasal airflow stimulates trigeminal mechanoreceptors that modulate ventilatory drive and pharyngeal dilator tone (Kubin, 2016). Reduced nasal airflow, whether due to structural narrowing or inflammation, decreases this sensory input and promotes upper airway collapse during sleep. This reflex-mediated link demonstrates that maintaining nasal airflow integrity supports airway stability, independent of direct anatomic changes.

### 3.3 Nasal obstruction, sleep architecture, and inflammation

Evidence from sleep studies and large population datasets supports that nasal obstruction disrupts sleep microstructure. Lavie et al. (1981) reported that individuals with allergic rhinitis exhibited a tenfold increase in microarousals compared with controls. McLean et al. (2005) demonstrated that nasal decongestant therapy improved sleep efficiency from 73 to 85% and increased deep and REM sleep durations. Chronic mouth breathing resulting from nasal obstruction elevates anatomical dead space by up to 40% and augments overnight hypercapnia, perpetuating sympathetic activation and arousal (Suzuki et al., 2015). Persistent turbulent airflow across narrowed nasal segments further promotes mucosal inflammation and congestion, creating a self-reinforcing cycle of congestion and resistance.

## 4 Nasal symptoms and CPAP tolerance

### 4.1 Prevalence and clinical impact

The panel then discussed nasal symptoms associated with CPAP therapy (Brimioulle and Chaidas, 2022; Chaidas et al., 2022). Despite decades of technological advancement, global CPAP adherence remains near 50%. Nasal symptoms rank among the most frequent reasons for CPAP intolerance (Brimioulle and Chaidas, 2022; Chaidas et al., 2022). Approximately 30%−50% of users experience nasal dryness, congestion, or rhinorrhea during the early phase of treatment. A systematic review of 63 studies found that lower nasal resistance and wider cross-sectional area were significantly associated with greater CPAP tolerance and longer nightly use (Brimioulle and Chaidas, 2022). In contrast, patients with unrecognized nasal obstruction were more likely to discontinue CPAP within the first 3 months of initiation. In some cases, nasal symptoms can actually improve with PAP therapy since heated humification can reduce nasal congestion if reactive hyperemia resolves with PAP treatment (Kline and Carlson, 1999). In clinical practice, nasal congestion can present as “pressure intolerance,” where CPAP pressure amplifies the feeling of nasal resistance despite correct device settings. This sensory feedback loop underscores the need to identify and treat nasal obstruction concurrent with CPAP use.

The discussion also highlighted the importance of patient preference in mask selection and the need to address underlying nasal obstruction before changing masks. A point was emphasized that a patient being randomized to a mask (e.g., full face mask) is very different from a patient choosing a particular mask (de Andrade Xavier et al., 2022; Genta et al., 2025; Schorr et al., 2012). In general, patient preferences are key to driving long term adherence and thus shared decision making was emphasized as a strategy to optimize adherence. One author (AM) shared clinical experience where after repeated CPAP mask failures, patients were eventually found to have nasal obstruction which, when addressed, led to improved CPAP adherence. There was general agreement that sleep medicine physicians should work closely with their ENT colleagues to optimize clinical outcomes for OSA patients.

### 4.2 Topical therapy and humidification

The group also discussed the variable effectiveness of topical nasal steroids. In clinical experience, the distinction between vasomotor rhinitis and allergic rhinitis is not always made (Skirko et al., 2020).

Skirko et al. (2020) found that only a minority of patients using fluticasone show meaningful and sustained nasal symptom relief (Skirko et al., 2020). Ensuring daily use with proper spray angle and incorporating heated humidification are essential to prevent mucosal dryness and reactive hyperemia (Kline and Carlson, 1999). For vasomotor rhinitis, topical anticholinergics such as ipratropium may be preferable over steroids. In cases of anatomic resistance, medical therapy alone rarely normalizes flow, supporting surgical intervention when appropriate. Authors agreed that proper education and consistent use are crucial for minimizing nasal symptoms.

## 5 Surgical and minimally invasive interventions

### 5.1 Conventional nasal surgery

The group then discussed the impact of nasal surgery on CPAP requirements and patient adherence (Camacho et al., 2015). They reviewed two systematic reviews (Correa et al., 2024; Stewart et al., 2004), finding that while nasal surgery can reduce CPAP levels and improve sleep quality, nasal surgery does not consistently lower AHI scores. Various procedures were included in these assessments, including septoplasty, turbinate reduction, polypectomy and others (Correa et al., 2024; Stewart et al., 2004).

Multiple prospective and retrospective studies demonstrate that correcting structural nasal obstruction improves patient comfort and CPAP adherence. Poirier et al. (2014) and Zonato et al. (2006) reported a mean reduction of 2–3 cm H_2_O in CPAP therapy pressure requirements after septoplasty and turbinate reduction (Poirier et al., 2014; Zonato et al., 2006). Two additional studies also observed that CPAP acceptance increased from 42 to 93% after nasal surgery in previously intolerant patients (Iwata et al., 2020; Nakata et al., 2005). Average device use improved from 0.5 h to approximately 5 h per night once nasal obstruction was addressed (Azbay et al., 2016; Elwany et al., 2022). These results demonstrate that while nasal surgery may not lower AHI, it substantially enhances device acceptance and tolerance.

### 5.2 Nasal obstruction and HNS outcomes

The group discussed the relationship between NAO and outcomes of hypoglossal nerve stimulation (HGNS) for OSA. Some authors noted that while improving nasal patency can potentially enhance HGNS outcomes by stabilizing the airway and improving upper airway muscle tone, mouth opening during sleep remains a major challenge. One author (PS) shared that the STAR trial did not show a clear benefit of prior nasal surgery on HGNS outcomes, but that at his center concurrent septoplasty and turbinate reduction are performed at times with HGNS implantation if there is a clear anatomic obstruction with symptoms.

### 5.3 Temperature controlled radiofrequency (TCRF) remodeling

The group discussed the clinical trial data for temperature-controlled radiofrequency (TCRF) treatment. TCRF (VivAer^®^) delivers controlled thermal energy to the submucosal layer of targeted nasal structures, most commonly the nasal valve area, inferior turbinates, and septal swell body. The energy produces collagen denaturation followed by new collagen synthesis and contraction. This localized remodeling restores and stabilizes the internal nasal valve, reduces dynamic collapse, and reestablishes laminar airflow with minimal trauma.

The authors noted that it can be used as an alternative to surgery or as an adjunct to other treatments. While the procedure is safe, patients typically experience severe congestion for a week post-procedure, followed by crusting of the nasal mucosa that lasts at least a month. The lead author (AV) then presented results from three trials: Han et al. (2025) reported long-term outcomes from a randomized controlled trial in 108 subjects. The authors demonstrated a mean symptomatic improvement of 45 points in the NOSE score and a decrease (i.e., improvement) 8.8 points after 12 months in daytime sleepiness (Han et al., 2025). Results were sustained to 3 years. Two additional single arm studies demonstrated durable improvements in nasal symptoms and sleep subscores for 3–4 years with minimal adverse events (Han et al., 2025; Jacobowitz et al., 2022; Yao et al., 2025). (Brehmer et al. 2019) also found a 25-point reduction in Snore Outcome Survey scores by 90 days post-procedure (Brehmer et al., 2019). Collectively, these studies confirm that TCRF provides efficient, office-based restoration of valve function and meaningful improvement in sleep-related quality of life. While other methods for turbinate reduction alone are options, no head-to-head comparison of inferior turbinate reduction alone vs. concurrent treatment of the swell body or nasal valve has been conducted to-date.

## 6 Nasal patency, mouth breathing, and sleep physiology

The group discussed the relationship between NAO, mouth breathing, and sleep quality (Lam et al., 2006; Rhee et al., 2010). Mouth breathing in OSA increases work of breathing by approximately 2.5-fold and reduces minute ventilation (Georgalas, 2011; McNicholas, 2008). Author (OJ) presented data from multiple studies demonstrating that mouth opening lowers upper airway muscle tone and raises critical closing pressure (Pcrit), and that these effects increase pharyngeal collapsibility (Ayuse et al., 2004; Basner et al., 1985; Meurice et al., 1996). Restoring nasal patency reduces oral airflow, shortens desaturation events, and improves sleep architecture (Varendh et al., 2018). Habitual mouth breathers may require behavioral retraining post-surgery, although most patients demonstrate improved oxygenation and enhanced CPAP tolerance after nasal intervention (Iwata et al., 2020; Nakata et al., 2005). The group concurred that identifying the correct patient phenotype is crucial for determining who might benefit from nasal surgery to improve sleep outcomes.

## 7 Nasal optimization and CPAP adherence

The group discussed the relationship between nasal resistance and CPAP adherence. Persistent structural nasal obstruction can undermine effective CPAP use. Population-level monitoring shows that only 55% of users achieve ≥ 4 h of use on ≥ 70% of nights (Weaver and Grunstein, 2008). In case series studies, patients intolerant to PAP were able to successfully use PAP after nasal surgery, associated with decreased nasal resistance (Nakata et al., 2005; Zonato et al., 2006). After nasal intervention, studies have demonstrated mean CPAP usage increases by approximately 1.5 h nightly and adherence rates rise by 17% (Poirier et al., 2014). Such improvements in PAP use can translate into clinically meaningful improvements, including modest reductions in hypertension incidence and cardiovascular events among adherent OSA patients (Bazzano et al., 2007; Marin et al., 2005). However, whether nasal intervention–associated improvements in adherence translate into similar long-term outcomes remains unknown and warrants further study. Despite the limited evidence on long-term outcomes, incorporating nasal assessment in CPAP management using NOSE scales, modified Cottle maneuver, and thorough nasal exams permits early identification of treatable obstruction. Collaboration between sleep physicians and otolaryngologists ensures comprehensive management, stabilizing CPAP use and improving long-term efficacy. In summary, the panel agreed that nasal assessment and optimization should be viewed as integral components of comprehensive OSA management. Clinical evidence consistently demonstrates that improved nasal airflow enhances sleep stability, comfort with positive airway pressure therapy, and long-term PAP adherence. Table 1 summarizes the key expert consensus positions and supporting evidence related to nasal physiology, CPAP efficacy, and interventional strategies.

**Table 1:** Summary of expert consensus recommendations on nasal optimization in OSA.

Topic	Consensus position	Supporting evidence	Practical implication
*Impact of nasal airway obstruction on sleep quality*	Nasal obstruction is associated with increased microarousals, reduced REM and deep sleep, and heightened sympathetic tone.	Lam et al., 2006; Rhee et al., 2010; Suzuki and Tanuma, 2020; Wakayama et al., 2016; Chang et al., 2023; Lavie et al., 1981; McLean et al., 2005; Varendh et al., 2018; Georgalas, 2011	Comprehensive OSA evaluation should include nasal airflow assessment to identify reversible causes of elevated resistance. Treating nasal obstruction can improve sleep architecture independent of AHI reduction.
*Nasal resistance and PAP efficacy*	High nasal resistance elevates therapeutic pressures, reduces comfort, and impedes adherence to CPAP.	Brimioulle and Chaidas, 2022; Camacho et al., 2015; Poirier et al., 2014; Zonato et al., 2006; Chang et al., 2023	Optimizing nasal patency enhances mask tolerance, allows lower CPAP pressures, and improves nightly use.
*Conventional nasal surgery*	Septoplasty, turbinate reduction, and related procedures improve nasal airflow and CPAP adherence, with modest changes in AHI but clinically meaningful symptom improvement.	Camacho et al., 2015; Poirier et al., 2014; Elwany et al., 2022	Surgical correction should be considered in CPAP-intolerant patients with documented structural obstruction.
*Minimally invasive approaches*	Office-based temperature-controlled radiofrequency (TCRF) therapy or similar procedures effectively remodel nasal structures to restore physiological airflow with low morbidity.	Jacobowitz et al., 2019; Silvers et al., 2021; Han et al., 2022; Jacobowitz et al., 2022; Pritikin et al., 2023; Yao et al., 2021	Provides a viable option for patients with nasal valve dysfunction refractory to medical therapy or when surgery is less desirable.
*Integration into multidisciplinary OSA care*	Coordination between otolaryngology, sleep medicine, and pulmonology is essential for identifying and treating nasal contributors to PAP intolerance.	Chang et al., 2023; Weaver and Grunstein, 2008	Routine nasal evaluation before and during PAP therapy can increase long-term adherence and treatment success.

## 8 Future directions

The meeting focused on exploring potential study designs to evaluate the effectiveness of nasal surgery in patients with OSA. Evidence consistently supports improving nasal function to enhance CPAP outcomes. Nevertheless, conducting prospective randomized trials to evaluate these relationships remains challenging due to the difficulty of isolating nasal influences from behavioral and lifestyle confounders.

Participants outlined a prospective, multicenter observational framework with embedded randomized elements to quantify changes in CPAP adherence, pressure requirements, sleepiness, and nasal resistance following valve intervention. Mechanistic sub-studies using rhinomanometry and acoustic rhinometry were proposed to correlate physiologic changes with adherence gains. Phenotype-based stratification was emphasized to identify patients most likely to benefit.

The group discussed three potential indications for improving NAO:

1) Patients with daytime symptoms who report congestion and/or impaired daily activities or exercise. In patients who are refractory to medical therapy, the use of surgical procedures was generally recognized as indicated.
2) A patient with snoring and poor sleep quality can sometimes respond well to improvements in nasal patency. The group felt that additional data are required, but conceptually, relief of NAO may be beneficial for these patients. In some cases, patients with mouth breathing may fall into this category.
3) For OSA patients struggling with CPAP, a subset of these patients will respond well to improvements in nasal patency. Many factors can drive a lack of adherence to therapy, including psychosocial and other factors. Among patients with poor adherence to PAP therapy who have concomitant NAO, improving nasal patency is likely to yield long-term improvements in PAP adherence. However, the need for more randomized trials in this area was acknowledged.

## 9 Discussion and conclusion

Nasal airway obstruction directly affects sleep quality, physiological breathing patterns, and tolerance of PAP therapy. Untreated obstruction undermines both adherence and perceived sleep quality, independent of changes in apnea–hypopnea index or oxygen saturation metrics. Restoration of nasal patency should be recognized as an essential component of comprehensive airway optimization rather than a comfort-based adjunct. Minimally invasive technologies such as TCRF treatment provide safe and efficient correction of structural nasal resistance, expanding the therapeutic window for patients who might otherwise discontinue PAP therapy.

The panel concluded that improving nasal airflow has the potential to yield benefits that extend beyond the nose itself. Enhanced patency could facilitate more stable inspiratory pressures, reduce turbulence-induced mucosal inflammation, and restore normal reflex activation of pharyngeal dilator muscles. These physiological effects if sustained correspond to greater CPAP comfort, and longer nightly use. Thus, in theory improving nasal patency could yield measurable improvements in cardiovascular markers linked to therapy adherence. Even modest gains in nightly usage on the order of an additional hour and a half may translate into clinically meaningful decreases in hypertension incidence and reduced cardiovascular risk. From a practice standpoint, incorporating nasal evaluation into standard OSA care provides an achievable opportunity to identify therapeutic targets to improve PAP adherence.

In conclusion, the consensus of the expert panel was that addressing nasal patency should become routine before and during PAP treatment to facilitate long-term tolerability and adherence. Proactive identification and treatment of nasal obstruction, supported by collaboration between otolaryngology and sleep medicine specialists, are likely to improve health outcomes. By reframing nasal optimization as a core component of airway management rather than an optional intervention, practitioners have the potential to enhance treatment success for patients with obstructive sleep apnea.

## Data Availability

The original contributions presented in the study are included in the article/supplementary material, further inquiries can be directed to the corresponding author.
